# Number line estimation and standardized test performance: The left digit effect does not predict SAT math score

**DOI:** 10.1002/brb3.1877

**Published:** 2020-10-19

**Authors:** Katherine Williams, Joanna Paul, Alexandra Zax, Hilary Barth, Andrea L. Patalano

**Affiliations:** ^1^ Department of Psychology Wesleyan University Middletown CT USA

**Keywords:** left digit effect, number line estimation, numerical cognition, standardized achievement tests

## Abstract

**Introduction:**

Recent work reveals a new source of error in number line estimation (NLE), the *left digit effect* (Lai, Zax, et al., 2018), whereby numerals with different leftmost digits but similar magnitudes (e.g., 399, 401) are placed farther apart on a number line (e.g., 0 to 1,000) than is warranted. The goals of the present study were to: (1) replicate the left digit effect, and (2) assess whether it is related to mathematical achievement.

**Method:**

Participants were all individuals (adult college students) who completed the NLE task in the laboratory between 2014 and 2019 for whom SAT scores were available (*n* = 227).

**Results:**

We replicated the left digit effect but found its size was not correlated with SAT math score, although it was negatively correlated with SAT verbal score for one NLE task version.

**Conclusions:**

These findings provide further evidence that individual digits strongly influence estimation performance and suggest that this effect may have different cognitive contributors, and predict different complex skills, than overall NLE accuracy.

## Graphical abstract

We replicate the recently discovered left digit effect in number line estimation. Unlike overall accuracy, the left digit effect is not correlated with math achievement, suggesting a different cognitive source.

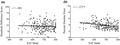

## 1 INTRODUCTION

Understanding numerical magnitudes is central to mathematical thinking. Skill in discrimination and judgment of relative magnitude is related to many aspects of mathematical competence including counting (Östergren & Träff, 2013), arithmetic (Moeller et al., 2011; Torbeyns et al., 2015), memory for numbers (Thompson & Siegler, 2010), fraction knowledge (Siegler et al., 2011), and standardized math achievement test performance (Ashcraft & Moore, 2012; Holloway & Ansari, 2009; Siegler et al., 2011). In fact, in studies of school readiness, early numerical magnitude skills have been found to more strongly predict later school success than other cognitive, attentional, or socio‐emotional skills (Duncan et al., 2007). Outside of the classroom, numerical magnitude skills predict more precise use of numbers by adults in the valuation of money (Schley & Peters, 2014), risk understanding (Patalano et al., 2020), and health decision making (Reyna et al., 2009). It is therefore not surprising that numerical magnitude skill has been identified as a diagnostic screening tool and as a crucial target for curricula and interventions aimed at improving mathematical thinking (Schneider et al., 2017). While most studies are largely correlational and thus do not demonstrate causal influence, they do suggest a critical link between magnitude processing and mathematical competence that warrants further investigation.

A mainstream view in numerical cognition is that we learn the meanings of numerals by mapping number symbols to approximate numerical quantities. According to this view, rather than processing numerals in a digital fashion, we access mental representations of their approximate analog quantities. One task that has been used to understand numerical magnitude skills is the number line estimation (NLE) task. In a typical NLE task, one is presented with a blank horizontal line labeled only with endpoints (e.g., 0 and 1,000) and asked to estimate the location of Arabic numerals on the line (e.g., “136”). This task is broadly used to train and assess skill in using and manipulating numerical magnitudes. The specific cognitive processes underlying performance on the NLE task have been debated, including whether age‐related improvements in performance are due to a loglinear to linear shift in the mapping of symbols to approximate magnitudes versus to changes in other task‐related skills such as proportion judgment and the use of additional reference points (Barth & Paladino, 2011; Barth et al., 2011; Cohen & Blanc‐Goldhammer, 2011; Siegler & Opfer, 2003; Siegler et al., 2009; Slusser & Barth, 2017; Slusser et al., 2013). However, a shared assumption has been that whatever the processes, it is the actual magnitudes of the target numerals themselves rather than their component digits that ultimately determine number line placements. The magnitudes of the individual digits that comprise the numerals (e.g., the “1” in “136”) have been seen as unimportant, and as having no influence on performance.

This view of NLE performance has been challenged by recent findings of Lai et al. (2018) who discovered a *left digit effect* in number line estimation. They found that estimates for numbers with different leftmost digits, but nearly identical magnitudes, were farther apart than is correct. For example, 602 was placed too far to the right of 599, though their magnitudes should be indistinguishable on a 0–1000 line. There were large effect sizes for both children and adults (*d*s > 1), whether the task was speeded or completed at one's own pace. Lai et al. (2018) used three‐digit numbers and only the leftmost (hundreds) digit contributed to this effect; three‐digit numbers with different tens place digits but similar magnitudes (e.g., 448 vs. 451) were not systematically placed in different locations. The leftmost digit was not *solely* driving performance in older children and adults: estimates were different for numbers with the same hundreds digit but distinguishable magnitudes (e.g., 801 vs. 899), indicating use of other digits to inform judgments as well. Younger children, however, produced indistinguishable estimates for these numbers, suggesting that their estimates are heavily influenced by the leftmost digit, but that left‐digit reliance may decrease with age. There were also noticeable individual differences across ages in that some individuals relied more heavily on leftmost digits than others.

The finding of Lai et al. (2018), while novel with regard to number line estimation, are broadly similar to findings in number comparison tasks in which digit‐level information has been shown to play an important role. For example, in price comparison studies, $5.00 is judged to be significantly more costly than $4.99, while $4.20 is not judged to be more costly than $4.19 (Beracha & Seiler, 2015; Lin & Wang, 2017; MacKillop et al., 2014; Manning & Sprott, 2009; Thomas & Morwitz, 2005, 2009). This left digit effect, where values with different leftmost digits are judged as farther apart in magnitude than those with the same left digit, has also been extended to the understanding of product nutritional information (Choi et al., 2019) and medical records (Olenski et al., 2020). Even in simple number comparison tasks (e.g., deciding whether 27 or 29 is larger) where the *distance effect* (i.e., faster response times for numerals that are farther apart from one another; Dehaene et al., 1990; Moyer & Landauer, 1967) has been attributed to comparisons of overall magnitudes, individual digits also matter. For example, there is a left digit effect in that response times are faster when comparison values have a different left digit (e.g., 49 vs. 51; Verguts & De Moor, 2005). There is also a *compatibility effect* whereby responses are faster when digit‐level information is compatible (e.g., for 42 vs. 57, 4 < 5 and 2 < 7) than when it is not (for 47 vs. 62, 4 < 6 but 7 > 2; Nuerk et al., 2001; Nuerk et al., 2011). It now seems that this type of digit‐level information may play a role in NLE as well.

There is a longstanding debate in the field regarding whether multidigit numbers are processed holistically (e.g., by converting “23” into a single holistic approximate magnitude; see Brysbaert, 1995; Dehaene et al., 1990), or in a componential fashion where each digit is independently mapped to an internal magnitude with multiple magnitudes contributing to task performance (Ganor‐Stern et al., 2007; Huber et al., 2016; Nuerk et al., 2011; Verguts & DeMoor, 2005), or both. The number comparison task, in particular, has played a large role in informing this debate. For example, Nuerk et al. (2001) have argued that compatibility effects indicate separate appraisal of and use of each digit's magnitude to perform the task (e.g., judging if 3 > 4 when comparing 33 to 41), consistent with a decomposition model. While a decomposition approach may appear on the surface to be more compatible with the left digit effect in NLE, to our knowledge neither type of model has yet been extended to the left digit effect in this task. Providing evidence in support of a particular model is not the goal of the present work, although we briefly consider these models further in the discussion.

A primary measure of performance on the number line estimation task is overall accuracy. *Percent absolute error* (PAE) reflects the difference between the actual placement and the correct location on the line relative to the length of the line used. Less common accuracy measures include percentage of correct responses, and percent variance explained (*R* ^2^) when the target numeral is used to predict estimates. These measures have been extensively linked to broader numerical competency, including children's counting ability (Östergren & Träff, 2013), performance on standardized math achievement tests (Booth & Siegler, 2008; Hoffmann et al., 2013; Holloway & Ansari, 2009; Schneider et al., 2009, 2018; Tosto et al., 2017) and adults’ numeracy (Peters & Bjalkebring, 2015; Schley & Peters, 2014). In a meta‐analysis, Schneider et al. (2018) found the relationship between PAE and achievement test score to be *r* ≈ 0.40 (mean age of 4–14 years old across studies). The relationship remains even after controlling for potentially confounding variables such as parental income and education, working memory, processing speed, and reading achievement (Bailey et al., 2014; Geary, 2011; Hansen et al., 2015; Hornung et al., 2014; Östergren & Träff, 2013; Zhu et al., 2017). This relationship motivates the use of the NLE task as a tool for training quantitative skills and for predicting future mathematics achievement. Because the left digit effect in NLE is newly identified, we do not yet know if measures of the left digit effect are similarly related to math achievement.

The study we present here has two goals. The first is to replicate the left digit effect in number line estimation. Towards this goal, we use two data sets. The first is a preexisting data set collected in our laboratory for unrelated purposes (reported in Patalano et al., 2020) prior to the discovery of the left digit effect in NLE. The data were collected using a speeded version of the NLE task identical to Lai et al. (2018) Experiment 1, where responses were required within a two‐second window. The second data set was collected across two recent studies in our laboratory involving a self‐paced version of the NLE task. In these studies, participants completed three blocks of NLE trials where the middle block constituted a feedback intervention for half the participants. We use only the first “baseline” block for purposes here (and report feedback findings elsewhere). This self‐paced version of the NLE task is similar to that used in Lai et al. (2018) Experiment 2 except with a larger set of target stimuli. These data sets are not selective; they reflect all NLE data collected in this laboratory in the past five years. However, given the goals of this work, we consider only the subset of participants from these data sets for whom SAT scores are also available.

As in Lai et al. (2018), we focus on *hundreds pairs*: three‐digit numbers with similar magnitudes but different leftmost digits (e.g., 799 vs. 801). Estimating these numbers to be in systematically different locations on the number line, with the larger placed to the right of the smaller, is evidence of a left digit effect. We also evaluate *fifties pairs* (e.g., 448 vs. 451) and *high–low pairs* (e.g., 498 vs. 401). We use these to establish, respectively, that the left digit effect is specific to pairs with different hundreds place digits (rather than also those pairs with different tens place digits), and that estimates are not driven by the hundreds place alone. We predict replication of the left digit effect across task formats. Given that NLE task variations are not our focus, we collapse over task version in reporting findings whenever doing so is warranted. This approach is supported by Lai et al. (2018) who obtained comparable measures of the left digit effect and of PAE when using the speeded versus the self‐paced version of the task.

Our second goal is to test whether the left digit effect is related to math achievement. This question is important for several reasons. First, the NLE task is often used to predict future math achievement, so it is valuable to know which measures are the best predictors. To this point, various NLE accuracy measures have been found to be equally good predictors of achievement test scores (Schneider et al., 2018), but we do not know if this finding extends to measures of the left digit effect. Second, the question has implications for training and instruction. Observing a relationship between the left digit effect and math achievement would be a first step in considering interventions aimed at reducing reliance on the leftmost digit. Third, we do not know why the left digit effect occurs and we do not know if it arises from the same cognitive sources as PAE (e.g., in part from imprecise mappings to mental magnitudes). If PAE and the left digit effect similarly predict math achievement (and more strongly than they predict verbal achievement), this would be suggestive evidence of common cognitive contributors.

Towards this goal, with participants’ consent, we obtained SAT verbal and math scores on file with the university. The SAT scores obtained were of one of two formats. The current SAT test format consists of one verbal and one math component, each with a score ranging from 200–800. However, prior to 2016, there were two verbal components and one math component, so we averaged these verbal scores to obtain a comparable measure to the present score. If the left digit effect is related to math achievement, it should be negatively correlated with SAT math score: individuals with a larger left digit effect should have lower SAT math scores. We had no predictions regarding SAT verbal scores other than that, if the left digit effect and PAE draw on the same skills, they should be similarly related to SAT verbal scores. There is some evidence of a weak relationship between NLE accuracy and reading skills in children (Namkung & Fuchs, 2016; Tosto et al., 2017), but most research has emphasized math skills. While the focus in the present work is on the left digit effect, we note that because there are few studies of the relationship between PAE and math achievement in adults (rather than children), replicating past findings with a college‐aged sample is also a valuable contribution.

## 2 METHOD

### 2.1 Participants

Included in the present study are data from *n* = 227 participants (144 women, 81 men, 1 undisclosed) who received course credit or monetary compensation for their participation. A power analysis indicated that at least 82 participants would be needed to detect a medium correlation of *ρ* = 0.30 with a power of 0.80 at the *α* = 0.05 level, so the sample exceeds this minimum. Participants consist of all individuals who completed the NLE task in the laboratory between 2014 and 2019 (*N* = 390 available), for whom SAT scores were available (*n* = 152 excluded). Of included participants, *n = *65 completed a speeded version of the NLE task (original findings published in Patalano el al. (2020); data collected prior to discovery of the left digit effect) and *n = *162 completed a self‐paced version of the task (used as a baseline measure in an unpublished study of feedback effects). Of included participants, *n* = 77 completed the pre‐2016 SAT test format and *n* = 150 completed the current SAT test format (these numbers largely overlap with whether the participant completed the speeded or self‐paced task version). Standardized test scores (SATs) on file with the university were obtained with participants’ written permission. Participant gender identity and native language(s) were also previously collected.

### 2.2 Ethical compliance statement

The study was approved by the University's Institutional Review Board. All participants gave their written informed consent to participate in the number line estimation task and to have their SAT scores obtained from the University for the purpose of this study.

### 2.3 Number line estimation task

This task assesses one's ability to identify the locations of numbers on a response line (Lai et al., 2018). Participants were seated in front of a computer and given written instructions. The task included presentation of the following 38 critical targets (falling on either side of nine hundreds boundaries and ten fifties boundaries): 47, 51, 98, 102, 147, 153, 199, 202, 249, 252, 298, 302, 349, 351, 398, 403, 449, 453, 499, 502, 547, 552, 597, 601, 647, 652, 699, 703, 747, 753, 798, 802, 848, 853, 899, 901, 949, and 953. Participants were instructed to select a position on the line (with a mouse click) to estimate the location of the given target numeral. Locations of mouse clicks were recorded and converted to numbers between 0 and 1,000, corresponding to the selected location on the response line.

#### 2.3.1 Speeded version

This task (identical to Lai et al., 2018, Exp. 1) was conducted using MATLAB software on a 14‐inch HP ProBook (with screen 32.4 cm in width × 19.2 cm in height; 1,366 x 768 pixels). Each trial consisted of a centered fixation rectangle (grey, 12.3 cm × 0.7 cm; 500 ms) followed by a stimulus screen displaying the target value (1.4 cm in height) in the center of the screen (e.g., “47”; 500 ms) followed by a response screen presenting a 12.3 cm horizontal line with vertical end lines (1.4 cm in length each) and labeled “0” on the left and “1,000” on the right (1,500 ms) appearing in a different computer‐generated pseudorandom screen location on each trial. The response line and end lines were 0.1 cm thick. When participants indicated with a mouse click where a number fell on the response line, a 2.0 cm long black vertical line (0.3 cm thick) appeared on the line in the selected location. A 1,000 ms pause separated trials. Each block (of two for a total of 76 trials) consisted of the 38 critical target values presented in a computer‐generated pseudorandom order (a different order for each participant). Two practice trials (different ones for each participant) were drawn randomly from target values. PAE was computed using all targets (also used to assess the left digit effect).

#### 2.3.2 Self‐paced version

This task (similar to Lai et al., 2018, Exp. 2) was conducted using PsychoPy software on a 21.5‐inch iMac (with screen 46 cm in width × 26 cm in height; 1,440 × 900 pixels). Each trial consisted of a target numeral (e.g., “47”; 1.8 cm in height) centered 8 cm above a 10‐cm horizontal line (that was 0.2 cm thick). The horizontal line had vertical end lines 1.0 cm in length (and 0.1 cm thick) and was labeled “0” on the left and “1,000” on the right. When participants indicated with a mouse click where each number fell on the line, a 1‐cm red vertical line appeared on the response line in the selected location. Besides the 38 critical boundary values, targets included 82 non‐boundary values (e.g., 235, 367, 411). Trials were self‐paced but response times were collected and participants were instructed to respond as quickly and accurately as possible (with a 500 ms pause separating trials). There were no practice trials. Participants completed one block of 120 trials with targets presented in a different computer‐generated pseudorandom order for each participant. (Participants who completed this version of the task completed three blocks of trials total. Only the first block is used here because feedback interventions were introduced in later blocks.) PAE was computed using non‐boundary targets (while boundary targets were used to assess the left digit effect).

### 2.4 SAT standardized test

The SAT (published by the College Board) is a standardized test commonly used for college admissions in the United States. The current format has two components: Math (58 questions assessing basic arithmetic, algebra, geometry, and trigonometry) and Evidence‐Based Reading and Writing (96 questions assessing reading comprehension, grammar, vocabulary in context, and editing skills) (with an optional essay component not reported to our university). The single Evidence‐Based Reading and Writing component replaces two separate components in the pre‐2016 test format (College Board, 2018): Critical Reading (67 questions assessing literal comprehension, vocabulary in context, and extended reasoning), and Writing (49 questions and a written essay assessing grammar usage and rhetorical skills). Scores on each component are reported on a scale ranging from 200 to 800. To create a single verbal score for pre‐2016 test takers, we averaged Critical Reading and Writing scores, resulting in one SAT verbal and one SAT math score per participant. (The pattern of findings is the same with use of Critical Reading or Writing score alone rather than the average of the two scores.)

## 3 RESULTS

NLE task version was not related to the size of the left digit effect (*t* < 1, *p *> .400; see later in Results for the measure of the left digit effect) or to PAE (*t* < 1, *p *> .500; similar to what was found across studies in Lai et al., 2018). It also did not moderate relationships with SAT scores (*F*s < 4, *p*s > .060). We first report all findings collapsed across versions although, in later exploratory analyses, we briefly revisit task version.

### 3.1 Number line estimation measures

Individual estimates were excluded if they differed from the group mean for a target value by more than 2 units of standard deviation (*M* = 3.9% of trials). To determine whether placements differed for paired numerals, we calculated difference scores (*placement of larger numeral – placement of smaller numeral*) for each hundreds, fifties, and high–low pair (e.g., the estimate for 302 minus the estimate for 298; Lai et al., 2018). At this point we excluded participants missing more than three hundreds, fifties, or high–low pairs (*n* = 11; 6 women, 5 men). Using all non‐boundary numerals, we calculated percent absolute error (PAE), a standard measure of overall accuracy error, as *PAE =* (*|actual placement – correct location|*)/1000 * 100. PAE was 3.9% (*SD* = 1.1; range = 1.9–7.2), similar to Lai et al. (2018). The mean response time for those completing the self‐paced version of the task was 2.9 s (*SD* = 1.3, range = 1.2–8.9).

For each individual, an average *hundreds difference score* was calculated by averaging individual difference scores for nine pairs: 98/102, 199/202, 298/302, 398/403, 499/502, 597/601, 699/703, 798/802, and 899/901 (the findings do not change with the exclusion of the pair that contains a two‐digit number: 98/102). An average *fifties difference score* was calculated by averaging difference scores for 10 pairs: 47/51, 147/153, 249/252, 349/351, 449/453, 547/552, 647/652, 747/753, 848/853, and 949/953 (the findings do not change with the exclusion of the pair that contains two‐digit numbers: 47/51). An average *high–low difference score* was computed by averaging difference scores for eight pairs: 102/199, 202/298, 302/398, 403/499, 502/597, 601/699, 703/798, and 802/899. Descriptive statistics are shown in Table 1.

**Table 1:** Descriptive statistics

	*M*	*SD*	Range	Skewness
Hundreds difference	20.6^a^	19.1	−24.4–82.1	+0.45
Fifties difference	0.1^b^	14.9	−45.1–41.9	+0.15
High–low difference	79.7^c^	19.2	21.4–139.6	–0.22
SAT verbal	685	65.0	455–800	–0.60
SAT math	691	73	480–800	–0.50

Note

*N* = 216.

^a^ Reliably greater than 0, indicating a left digit (hundreds) effect for hundreds pairs.

^b^ Not reliably different from 0, indicating no tens digit effect for fifties pairs.

^c^ Reliably greater than 0, indicating estimates were not based solely on hundreds digit.

### 3.2 Measuring the left digit effect

We first asked whether target numerals in each hundreds pair were placed in the same location on the line. If estimates are generated from the magnitudes of target numerals and component digits are irrelevant, the distance between these estimates should be approximately 0 because their magnitudes should be indistinguishable on a 0–1000 scale (e.g., 599 and 601 should be placed in approximately the same location). Evidence for a left digit effect comes from paired estimates being placed in very different locations on the line with the larger number placed to the right of the smaller number, leading to hundreds difference scores that are greater than 0. Hundreds difference scores (*M = *20.6) were reliably greater than 0, *t*(215) = 15.84, *p* < .001, *d* = 1.08 [95% CI: 18.0, 23.1]; the larger number in a pair was placed systematically too far to the right of the smaller number, on average. Further, we found that the 87% of participants had a hundreds difference score greater than 0 (and 70% had a score >10) indicating that most participants showed the effect.

Fifties difference scores (*M* = 0.1) were not reliably different from 0 (*t*(215) = 0.06, *p* = .955); for example, numbers like 147 and 151 were placed in approximately the same location on the line. This pattern demonstrates that the observed digit effect was indeed specific to the leftmost hundreds digit. If the tens digit were also heavily influencing estimates, for example, 151 would have been placed farther to the right of 147. High–low difference scores (*M* = 79.7) were reliably different from 0, *t*(215) = 61.08, *p* < .001, *d* = 4.16 [95% CI: 77.1, 82.3]. This is not a surprising result in adult participants given that these pairs were nearly 100 units apart, but it does indicate that hundreds place digits, while influential, were not solely responsible for estimates.

These findings replicate adult findings from Lai et al. (2018) and demonstrate that leftmost digits reliably influence performance. Difference scores were not related to gender identity or English as a native language as shown in Table 2.

**Table 2:** Pearson correlations between NLE measures and verbal and math scores

	PAE	NLE average difference scores		
		Hundreds	Fifties	High–low
Gender	+0.033	–0.020	+0.019	+0.017
English	–0.025	+0.034	–0.117	–0.018
SAT verbal	–0.171*	–0.142*	–0.060	+0.165*
SAT math	–0.272**	–0.061	–0.021	+0.092

Notes

Gender (0 = man, 1 = woman); English (0 = non‐native, 1 = native); for SAT verbal and math scores *r* = 0.549; **p < .001, *p < .05.

### 3.3 Relationship between left digit effect and SAT scores

**Figure 1:**
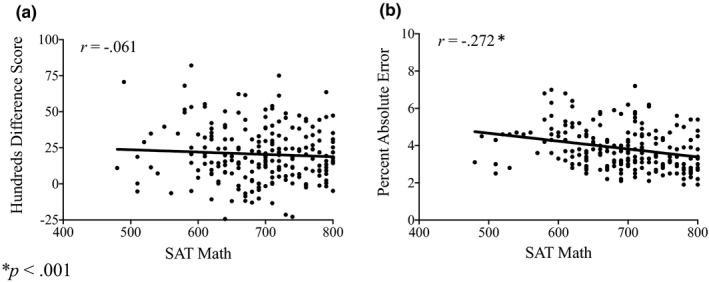
Scatterplots of the relationship between SAT math and (a) hundreds difference score and (b) percent absolute error (across task versions)

We next asked whether the left digit effect was related to formal math and verbal skills. See Table 1 for SAT descriptive statistics. As shown in Table 2 and in the scatterplot in Figure 1, counter to our predictions, there was no relationship between SAT math score and either hundreds difference score (*r*(214) = –0.061,*p* = .373) or high–low difference score (*r*(214) = 0.092,*p* = .179). There was, however, a weak but statistically significant relationship between SAT verbal score and hundreds difference score (*r*(214) = –0.142,*p* = .038) as well as high–low difference score (*r*(214) = 0.165,*p* = .015). In other words, participants with stronger verbal skills had a smaller left digit effect. We also conducted an exploratory analysis in which we re‐ran each correlation using the residuals after regressing each SAT score on to the other score. This allowed us to remove shared variance in order to assess whether the left digit effect is associated with skills unique to each test component (see Blatt et al., 1998, for approach). The pattern of findings remained the same. There were no statistically significant correlations involving residualized SAT math score and difference scores (|*r*|s < 0.11, *p*s > .100), the relationship between hundreds difference score and residualized SAT verbal score approached statistical significance (*r*(214) = –0.129, *p* = .059), and the relationship with high–low difference score was statistically significant (*r*(214) = 0.137, *p* = .044). In sum, counter to our predictions, there was no relationship between the left digit effect and SAT math score, but a weak negative relationship with SAT verbal score that cannot be attributed to shared variance between SAT scores.

### 3.4 Relationship between PAE and SAT scores

We also asked whether PAE was related to formal math and verbal skills, given the extensive past evidence that PAE is related to math achievement among children. Here we found that PAE was moderately correlated with SAT math score (*r*(214) = –0.272,*p* < .001); as predicted, individuals with higher SAT math scores had lower PAE (see Figure 1). There was also a weak correlation between PAE and SAT verbal score (*r*(214) = –0.171,*p* = .012). When we re‐ran the analyses using regression residuals, there remained a correlation between PAE and residualized SAT math score (*r*(214) = –0.213,*p* = .002) but no longer between PAE and residualized SAT verbal score (*r*(214) = –0.032,*p* = .637). These findings indicate that, consistent with our predictions, there is a relationship between PAE and SAT math scores. There is also a weak relationship with SAT verbal score that may be related to general cognitive skills associated with both SAT components. The correlation between PAE and SAT math score was less than in past studies (where *r* ≈ 0.40), but this difference may exist because the present study involved selective college students rather than school‐aged children.

### 3.5 Additional exploratory analyses

**Figure 2:**
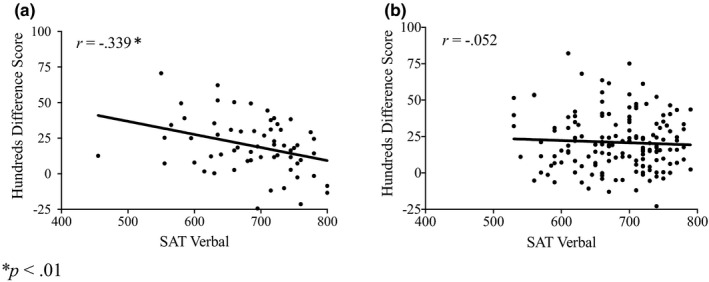
Scatterplots of the relationship between SAT verbal and hundreds difference score for the NLE task (a) speeded version and (b) self‐paced version

To better understand the unexpected relationship between SAT verbal scores and the left digit effect, we considered correlations as a function of NLE task version. In the speeded task, we found a reliable correlation between SAT verbal score and hundreds difference score (*r*(59)* = *–0.339, *p* = .007) and high‐low difference score (*r*(59)* = *0.320, *p* = .012); these were not present in the self‐paced task (|*r*|s < 0.100, *p*s > .100), as shown in Figure 2. These findings suggest that the relationship between the left digit effect and SAT verbal scores is driven largely by the speeded task, in which less skilled readers may have difficulty reading targets as quickly as is demanded by the task. In contrast, for the relationship between PAE and SAT math score, correlations were the same under speeded (*r*(153) = –0.265, *p* = .039) and self‐paced (*r*(153) = –0.271, *p* = .001) versions of the task. The left digit effect and SAT verbal score finding should be interpreted cautiously, as the earlier analysis of task version as a moderating variable did not reach statistical significance. It is also the case that because task version is largely confounded with SAT format, we cannot rule out the possibility that SAT format may moderate the correlation, although this strikes us as considerably less plausible given that the two SAT formats were highly similar and were intended to assess the same skills.

## 4 DISCUSSION

There are several major findings. First, the study replicates the finding of a left digit effect in adult NLE: numbers with different leftmost digits but similar magnitudes (e.g., 399 and 402) are estimated very differently. Consistent with previous work, a parallel pattern was not observed for numbers with similar magnitudes and the same leftmost digit (e.g., 348 and 351) suggesting that the estimation pattern observed depended specifically on the influence of the leftmost hundreds place digit. Not surprisingly for our adult sample, numbers with the same leftmost digit but very different magnitudes (e.g., 302 and 399) were estimated differently, indicating that estimates were not solely driven by the leftmost (hundreds) digit. The findings were again robust across speeded and self‐paced NLE task versions. They contribute to the growing body of evidence that numerical estimates are not solely driven by the overall magnitudes of the target numerals themselves. Rather, any account must also explain the influence of individual digit identity on performance.

While the study was not conducted to test models of number processing, we draw attention here to a recent model of number‐to‐quantity conversion developed by Dotan and Dehaene (2020). In this model, each digit is bound to a syntactic role (e.g., hundreds, tens; following McCloskey, 1992; McCloskey et al., 1985, 1986), and is weighted according to its role. Digit‐based quantities are then combined into a whole‐number quantity that, in the case of NLE, informs number line placement. This model is more flexible than purely holistic models (in which each multidigit numeral is typically assumed to have its own lexical entry), and posits access to weighted digit‐based magnitudes that might be used when task‐relevant (such as two‐digit number comparison in which it is often possible to respond accurately using only the leftmost digit), but also assumes the rapid construction of whole‐number estimates. Such a model may be a good candidate for understanding the left digit effect in number line estimation, perhaps as an overweighting of leftmost digits during the integration process (an account suggested by Thomas & Morwitz, 2005, but not in the context of this model), rather than as resulting from use of multiple digit‐level representations to perform the task.

The extension of the left digit effect finding beyond the context of Lai et al. (2018) is not considerable here, but it is noteworthy that one difference in the present procedure was the use of non‐boundary (e.g., 235, 411) as well as boundary (e.g., 199, 502) targets for the majority of participants. In the self‐paced version of the task, in addition to the 38 boundary targets, there were 82 non‐boundary targets. The addition of the latter made the goals of the study less transparent and increased the average distance between paired values by providing more intervening trials. If the left digit effect depends on the proximity of paired values, we would have expected it to be reduced with this procedure, but it remained robust, as in past work. The inclusion of a large number of non‐boundary values also makes it possible to compute PAE using only these values, thereby removing variance associated with the left digit effect that would otherwise be included. This use of non‐boundary values may be desirable to adopt broadly in light of the present findings.

A second major finding is that the pattern of correlations with SAT scores is, in fact, very different for PAE and for the left digit effect. For the former, consistent with past work on the link between estimation performance and formal math abilities (Schneider et al., 2018; Siegler et al., 2011), we found that PAE predicted SAT math score (which includes basic arithmetic, algebra, geometry, and probability) across NLE task versions. PAE explained about 7% of the variance in math achievement, which is less than the ~16% in past work but is still considerable given the present study involved high achieving students who opted to include SATs in their college application. In contrast, under neither task version was the hundreds difference score related to SAT math skills, suggesting that it may not arise from the same cognitive source. This finding also suggests that left digit effect and accuracy measures cannot be used interchangeably as predictors, and it offers no evidence that developing interventions to reduce the left digit effect (e.g., through instruction or accuracy feedback) might be an effective approach to improving numerical magnitude skills or SAT‐based math achievement.

A third major finding is that the degree to which individuals exhibited a left digit effect was, under some conditions, related to SAT verbal score (e.g., literal comprehension, vocabulary in context, grammar usage, and rhetorical skills). Specifically, in the speeded version of the NLE task, 11% of variance in SAT verbal scores was explained by hundreds difference scores. It is unlikely that less skilled readers adopt simplifying strategies of focusing exclusively on the leftmost digit, as numbers with the same hundreds digit but very different magnitudes (e.g., 801 and 899) were still estimated differently. However, given that both number and word comprehension have been proposed to involve similar constructive processes including parallel processing of individual units (digits and letters) and the creation of syntactic structures into which units are assigned (see Dotan & Dehaene, 2020; McCloskey et al., 1986), it may be that some readers perform these tasks less efficiently across types of content. Relatedly, working memory, specifically the ability to store information while executing processing operations, is a well‐established predictor of reading comprehension in adults (see Daneman & Merikle, 1996, for meta‐analysis), raising the possibility that working memory is important for the integration of digit information into overall magnitudes as well. Finally, interestingly, Tu and Pulig (2018) recently observed a larger left digit effect in pricing judgments for individuals who have a more analytic rather than a holistic thinking style and concluded that a lack of holistic thinking is one mechanism underlying the left digit effect. Their findings combined with the present ones suggest there may be multiple contributors to digit‐based processing of numerals.

To our knowledge, the present study is the first to consider formal verbal and math skills in their relationship to the left digit effect, setting the stage for future studies. It will be important to build on present findings through use of measures that tap into more specific verbal and math skills (e.g., phonemic awareness, sight word recognition, approximate number system acuity, place‐value understanding, etc.), through introduction of variables related to cognitive style (e.g., analytic vs. holistic thinking), and through the use of samples with an even wider range of SAT test component scores. Other directions include considering the left digit effect in a developmental context, such as in its relationship to the development of specific verbal and math skills in childhood and considering the effect in a crosslinguistic context. If the order in which digits are processed contributes to the effect, crosslinguistic differences may emerge as a result of the order in which individual digits in number words are read; for example, “28” is “twenty‐eight” in English, but “acht en twintig” (eight and twenty) in Dutch (Savelkouls et al., 2020).

## 5 CONCLUSIONS

The left digit effect has been observed across a range of tasks and contexts including price judgments, number comparison, and number line estimation. Recent evidence reveals that even physicians show a left digit effect with regard to patient ages: Based on seven years of Medicare data, cardiac bypass surgery is less likely to be recommended for patients who have just turned 80 compared to those well into being 79 years old (Olenski et al., 2020), while there is no difference for individuals on either side of 78 years old (for which the leftmost digit does not change). Such findings speak to important consequences of the left digit effect for behavior and even for skilled decision making. Ongoing work addresses whether the left digit effect in number line estimation predicts the left digit effect exhibited in more complex judgment tasks, and also whether it is possible to use instruction and feedback to reduce the effect. Even if the left digit effect in number line estimation is not a predictor of math achievement, there are compelling reasoning for working to better understand the contributors to this effect and how to reduce overreliance on the left digit through education.

## CONFLICT OF INTEREST

All authors declare that they have no competing interests.

## AUTHOR CONTRIBUTIONS

H.B., A.P., and K.W. developed the study concept and design. Data collection was performed by A.P. and members of her laboratory (including K.W., A.Z., and J.P). K.W., A.Z., and J.P performed data coding and analyses. K.W. drafted the manuscript. All authors provided revisions and approved the final version of the paper for submission.

### Peer Review

The peer review history for this article is available at https://publons.com/publon/10.1002/brb3.1877.

## Data Availability

Data used in this study are openly available at https://doi.org/10.17605/OSF.IO/UAZR5
